# Effects of spray drying, freeze drying, and vacuum drying on physicochemical and nutritional properties of protein peptide powder from salted duck egg white

**DOI:** 10.3389/fnut.2022.1026903

**Published:** 2022-10-19

**Authors:** Tianyin Du, Jicheng Xu, Shengnan Zhu, Xinjun Yao, Jun Guo, Weiqiao Lv

**Affiliations:** ^1^College of Biological and Food Engineering, Anhui Polytechnic University, Wuhu, China; ^2^College of Engineering, China Agricultural University, Beijing, China

**Keywords:** salted duck egg white, protein peptide powder, freeze drying, physicochemical and nutritional properties, spray drying

## Abstract

Salted duck egg white contains many kinds of high quality protein, but it is often discarded as food factory waste because of high salinity and other reasons. The discarded salted duck egg white not only causes a waste of resources, but also causes environmental pollution. Using salted duck egg white as raw material, this study was completed to investigate the effects of three drying methods including freeze drying, vacuum drying, and spray drying on physicochemical and nutritional properties of protein powder from salted duck egg white. The results showed that the solubility, foaming and foaming stability, emulsification and emulsification stability of the protein peptide of salted duck egg white decreased to different degrees after drying. The scavenging rates of freeze-dried samples for superoxide anion, hydroxyl radical, and 1,1-Diphenyl-2-picrylhydrazyl (DPPH·) reached 48.76, 85.03, and 80.17%, respectively. Freeze drying had higher scavenging rates than vacuum drying and spray drying. The results of electron microscopy showed that freeze-drying had the least effect on the structure of protein peptide powder of salted duck egg white. The purpose of this experiment was to provide theoretical guidance and technical support for industrial drying of salted duck egg white protein solution.

## Introduction

The vast majority of duck eggs are made into salted duck egg products and are consumed by the public. Among them, salted duck egg yolk is favored by consumers for its unique taste and is used in traditional Chinese delicacies such as moon cakes (1, 2). In the production of salted duck egg yolk, the salted duck egg white has not been properly used because its salt content is as high as 7–12% (3, 4). Discarded salted duck egg white not only causes a waste of resources but also causes environmental pollution (5, 6). Therefore, the comprehensive utilization of salted duck egg white is an urgent problem to be solved in food factories. The team of researchers previously compared the enzymatic hydrolysis effects of papain, trypsin, acid protease, alkaline protease, neutral protease, and flavor protease on salted duck egg white. The results showed that papain was the most suitable protease for enzymatic hydrolysis of salted duck egg white, and the supernatant of enzymatic hydrolysis of salted duck egg white was finally obtained.

The main physicochemical and nutritional properties of egg white protein peptide powder include solubility, foaming, emulsification, and antioxidant (7, 8). The processing properties of protein include solubility, foaming, and emulsification. These functional properties play an important role in food processing and storage (9, 10). Free radicals are abundant in the human body. Under normal circumstances, free radicals in the body are in a dynamic balance of production and removal (11, 12). However, when free radicals are produced too much or removed too slowly, they will cause damage to the body. Excessive free radicals lead to a series of diseases such as aging and carcinogenesis (13, 14). Free radicals mainly include superoxide anion (O_2_-·), molecular hydrogen peroxide, hydroxyl radical (·OH), and singlet oxygen (O_2_·). The free radical scavenging ability test is considered as the most important method to evaluate the antioxidant activity of bioactive peptides (15). Studies have shown that the active peptide in salted duck egg white can effectively remove the excess free radicals in the body, protect the body from free radical attack, and prevent free radical induced disease or aging (16). Therefore, it is of great significance to study the processing characteristics and antioxidant activity of protein peptide powder of salted duck egg white.

In this experiment, the supernatant of enzymatic hydrolysis of salted duck egg white was processed by spray drying, freeze drying, and vacuum drying to prepare protein peptide powder of salted duck egg white. The effects of three drying methods on the main physicochemical and nutritional properties of egg white protein peptide powder were studied. The experimental results can provide theoretical reference and technical support for the preparation of protein peptide powder of salted duck egg white, and have important practical significance for the efficient development and utilization of salted duck egg white.

## Materials and methods

### Raw materials

The salted duck egg white was provided by Anhui Tianhe Food Co., LTD. The salted duck egg white was placed in the refrigerator at −20°C and thawed at room temperature before use. Papain was purchased from Beijing Hongrun Baishun Technology Co., LTD. All the other reagents were analytically pure.

### Preparation of protein peptide powder of salted duck egg white

**Figure 1:**
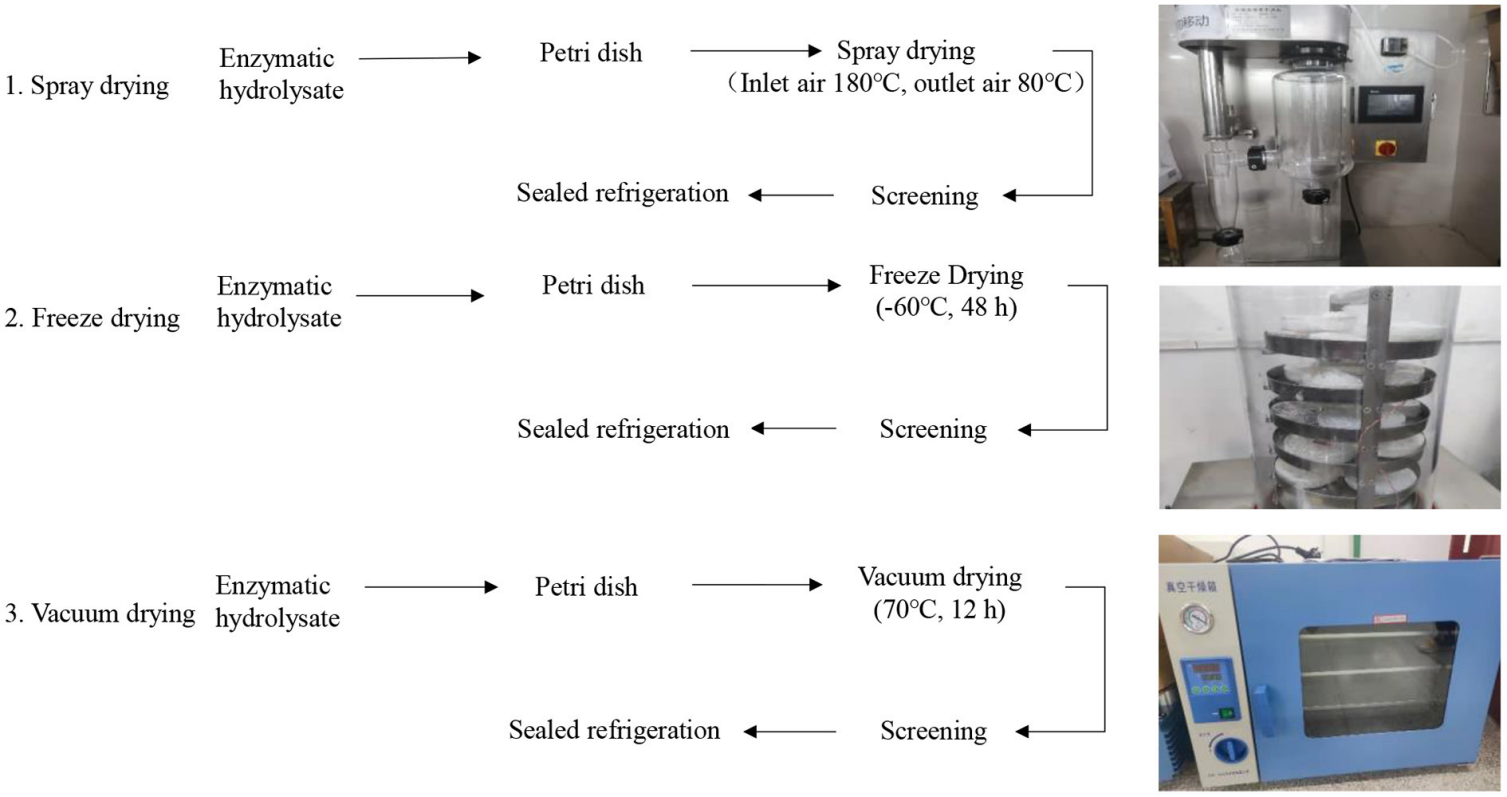
Preparation process of protein peptide powder of salted duck egg white by three methods.

Protein peptide powder was prepared from enzymatic hydrolysate of salted duck egg white by spray drying, freeze drying, and vacuum drying. According to the previous research results of the research team, the optimal conditions for spray drying were the inlet air temperature of 180°C and outlet air temperature of 80°C. The optimal conditions for freeze-drying were −60°C for 48 h. The best vacuum drying conditions were 70°C, vacuum degree 100 Pa, and vacuum drying for 12 h. The process flow of the three drying methods was shown in Figure 1.

### Determination of solubility of protein peptide powder from salted duck egg white

The solubility of protein peptide powder was determined according to the Gao method and adjusted appropriately (17). The protein peptide powder was prepared in a solution with a concentration of 10 mg/mL. The prepared solution was stirred by a magnetic stirrer for 30 min at room temperature and centrifuged at 6797 × g for 15 min. The nitrogen content in the supernatant and protein peptide solution before centrifugation was determined by the Kjeldahl method. The calculation formula of protein peptide solubility of salted duck egg white is as follows:

Solubility (%) = A/B × 100%················· ·················

Where A is the nitrogen content in supernatant and B is the nitrogen content in protein peptide solution before centrifugation.

### Determination of foaming of protein peptide powder from salted duck egg white

The foaming of protein peptide powder was determined according to the Eljoudi method and adjusted appropriately (18). The protein peptide powder was prepared in a solution with a concentration of 10 mg/mL. Then, 50 mL of peptide solution was taken and bubbled with a high-speed stirrer at the speed of 8603 × g for 5 min, and immediately pour into the measuring cylinder to read the volume number of foam recorded as V_0_. Then, the protein peptide solution was stationary for 30 min, and the volume number of foam was read again as V_30_. The calculation formula of protein peptide foaming capacity and foaming stability of salted duck egg white were as follows:

Foaming capacity (%) = $\frac{V_{0}-50}{50}$ × 100%··················

Foam stability (%) = $\frac{V_{30}-50}{V_{0}-50}$ × 100%······················

Where V_0_ is the volume number of foam of 0 min and V_30_ is the volume number of foam of 30 min.

### Determination of emulsification of protein peptide powder from salted duck egg white

The foaming of protein peptide powder was determined according to the Li method and adjusted appropriately (19). The protein peptide powder was prepared to a solution with a concentration of 1 mg/mL. Then, 30 mL of protein peptide sample solution at a concentration of 1 mg/mL was mixed with 10 mL soybean salad oil. The mixed solution was homogenized at 8603 × g for 15 min. At the end of homogenization, 50 μL of the emulsion was immediately sucked through a straw from the bottom of the solution and added to 5 mL of 0.1% SDS solution. The absorbance (A_0_) of the solution was measured at 500 nm using a spectrophotometer, which is the emulsification of the solution. Then the solution was allowed to stand for 10 min. Again, 50 μL of emulsion was absorbed from the bottom of the solution and added to 5 mL of 0.1% SDS solution. The absorbance (A_10_) was measured at 500 nm using a spectrophotometer. The calculation formula of emulsion stability is as follows:

Emulsification stability (%) = $\frac{A_{0}\;\times \;10}{A_{0}-A_{10}}$ ······················

Where A_0_ is the absorbance of the solution of 0 min, which is the emulsification of the solution. and A_10_ is the absorbance of the solution of 10 min.

### Determination of protein peptide powder from salted duck egg white by scanning electron microscope

A small amount of protein peptide powder was glued to the double-sided tape on the scanning electron microscope sample table. After coating with an ion sputtering apparatus (BAL-TEC, SCD 005), the samples were observed under a scanning electron microscope (FEI-Quanta 200). The powder structure of protein peptide powder of salted duck egg white prepared by three different drying methods was analyzed.

### Determination of superoxide anion (O_2_-·) scavenging ability of protein peptide powder from salted duck egg white

In this study, based on the study of Misak et al. (20), the pyrogallol autoxidation method was used to determine the superoxide anion scavenging activity of protein peptide powder from salted duck egg white. Deionized water was added to the protein peptide powder of salted duck egg white and prepared into a 5 mg/mL sample solution. Reagent A: Sample solution (0.2 mL) was added to test tube A. Then, 4.5 mL of Tris-HCl buffer (pH 8.2, 50 mmol/L) and 4 mL of distilled water were added to the tube. After shaking well, the test tube was placed in a 37°C incubator for 20 min. Reagent B: Pyrogallol solution (0.5 mL, 3 mmol/L) was added to test tube B. The tube B was kept warm in a 37°C incubator for 10 min. Reagents A and B were rapidly mixed and the absorbance was measured at 30 s intervals at 325 nm. In total, the absorbance value of 3 min was measured, and the regression line was made to find the slope K_1_ of the absorbance change. The blank control was carried out with distilled water instead of sample liquid, and the absorbance change slope K_0_ was obtained by the same method. The samples of each group were measured three times and then the average value was taken. The calculation formula of superoxide anion (O_2_-·) scavenging ability of protein peptide powder is as follows:

Scavenging ability (%) = $\frac{K_{0}-K_{1}}{K_{0}}$ × 100% ······················

Where K_0_ is the change slope of absorbance in the blank group within 3 min and K_1_ is the change slope of absorbance in the sample group within 3 min.

### Determination of hydroxyl radical scavenging ability of protein peptide powder from salted duck egg white

This test referred to the method of Prasad and Mishra (21), for determining the hydroxyl radical scavenging ability of protein peptide powder from salted duck egg white, and made appropriate modifications. Deionized water was added to the protein peptide powder of salted duck egg white to form a sample solution of 5 mg/mL. Then, 0.4 mL phosphate buffer (pH 7.4, concentration 200 mmol/L), 0.6 mL o-dinitrogen solution (5 mmol/L), and 0.6 mL ferrous sulfate solution (5 mmol/L) were added to the test tube. The test tube was shaken to mix the mixture well. After shaking, 2 mL of sample solution (5 mg/mL) and 0.4 mL of hydrogen peroxide (mass fraction 0.1%) was added to the mixture. The test tube was shaken again to mix the mixture well. The mixture was placed in a constant temperature incubator at 37°C for 60 min. Then the mixture was removed and the absorbance A_1_ was measured at 536 nm. Using deionized water instead of sample solution, the absorbance value A_0_ was measured by the same method as above. Using deionized water instead of sample solution and hydrogen peroxide solution, the absorbance A_2_ was measured by the same method. The samples of each group were measured three times and then the average value was taken. The calculation formula of hydroxyl radical scavenging ability of protein peptide powder is as follows:

Hydroxyl radical scavenging ability (%) = $\frac{A_{1}-A_{0}}{A_{2}-A_{0}}$ × 100% ······················

Where A_0_ is the absorbance measured by deionized water instead of sample liquid; A_1_ is the absorbance measured by sample liquid; A_2_ is the absorbance measured by deionized water instead of sample liquid and hydrogen peroxide.

### Determination of 1,1-diphenyl-2-picrylhydrazyl (DPPH·) scavenging ability of active peptide from salted duck egg white

DPPH· free radical scavenging ability was determined by a visible spectrophotometer and detection kit. Deionized water was added to the protein peptide powder of salted duck egg white to form a sample solution of 50 g/L. Then, 100 μL of sample solution and 900 μL of extract solution provided by the kit were added to a test tube for sample processing. The mixture was mixed with a vortex oscillator and centrifuged at 8603 × g for 15 min. After centrifugation, the supernatant was placed on ice to be tested. The spectrophotometer was preheated for more than 30 min, the wavelength was adjusted to 515 nm, and the wavelength was adjusted to zero with absolute ethanol.

The reagents in Table 1 were added to 1.5 mL EP tubes. The mixture was mixed with a whirlpool oscillator, and the absorbance value was measured at 515 nm after standing at room temperature (25°C) for 30 min in the dark. Each measurement tube shall be provided with a control unit. Blank tube only needs to be measured 1~2 times.

**Table 1:** Proportion of reagents in EP tubes.

**Reagents (μL)**	**Blank tube**	**Test tube**	**Control tube**
Supernatant	–	25	25
Reagents three	–	–	–
Extract solution	25	–	–
Reagents one	–	–	975
Working liquid	975	975	–

The calculation formula of DPPH· scavenging ability of protein peptide powder is as follows:

DPPH· scavenging ability = $\frac{A_{0}-\left(A_{1}-A_{2}\right)}{A_{0}}$ × 100% ······················

Where A_0_ is the absorbance value of the blank tube; A_1_ is the absorbance value of the test tube; A_2_ is the absorbance value of the control tube.

### Statistical analysis

The SPSS 20.0 software (IBM, Chicago, IL, USA) was used for the ANOVA of the samples in the study. Significant differences were determined by Duncan's multiple comparison test (*P* < 0.05).

## Results and discussion

### Solubility analysis under three drying methods

Solubility is an important indicator of protein applicability. Most of the functional properties of proteins are closely related to their solubility (22). The solubility of a protein is affected by conditions such as pH, ionic strength, temperature, and solvent type. The solubility of protein decreases with the extension of heating time (23).

**Figure 2:**
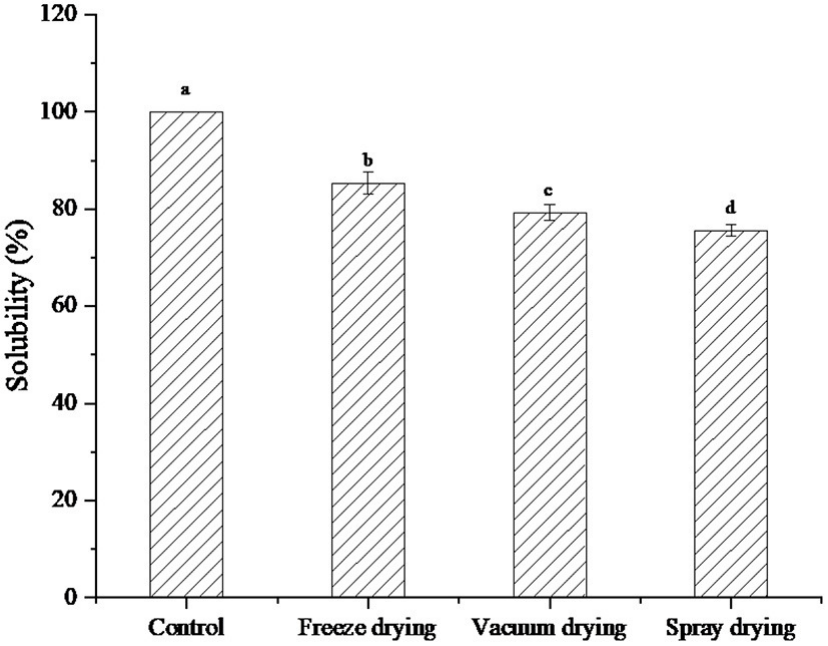
Solubility of protein peptide powder by three dry methods. Different letters express significant differences (*p* < 0.05).

The solubility of protein peptides under the three drying methods was shown in Figure 2. After drying in different ways, the solubility of protein peptides from salted duck egg white decreased to different degrees. The solubility of freeze-dried protein peptide samples was significantly higher than that of vacuum-dried and spray-dried samples. Li et al. (24) found that heating temperature can significantly affect the solubility of sarcoplasmic proteins. With the increase in heating intensity, the protein structure is depolymerized and aggregated. This cross-linking phenomenon increased the hydrophobic groups in sarcoplasmic proteins, resulting in enhanced surface hydrophobicity of protein molecules. The lowest solubility was found in the spray-dried samples. This may be because, in the process of spray drying, high temperature caused the structure of protein peptide molecules to unfold, and the hydrophobic groups originally embedded in the molecules were exposed to the surface of the molecules. The exposure of hydrophobic groups attenuated the interaction with water molecules, resulting in a significant decrease in solubility. However, due to the low temperature of freeze-dried samples, the protein peptides still maintained good solubility.

### Foaming analysis under three drying methods

**Figure 3:**
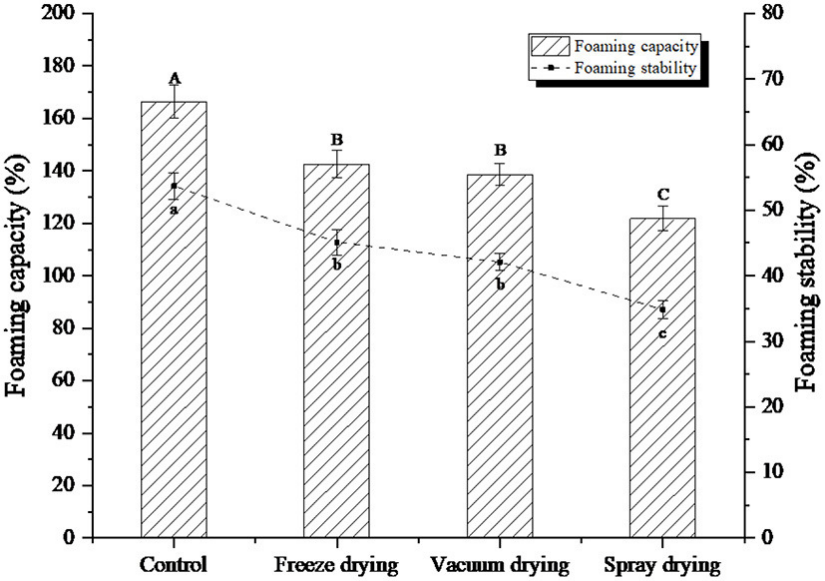
Foaming capacity and foaming stability of protein peptide powder by three dry methods. Different letters express significant differences (*p* < 0.05).

The foaming ability and foam stability of proteins are closely related to the hydrophobic amino acid residues on the molecular surface (25). Foams are generally two-phase systems in which the gas is distributed in a liquid phase. Since the gas phase is separated from a continuous phase, it has high surface tension and an unstable system, so the bubbles are easy to accumulate and fracture (26). The foaming property and foam stability changes of protein peptides under three drying methods were shown in Figure 3. As can be seen from the figure, the foaming value of the original protein peptide solution was 166.32%, and the foam stability value was 53.68%.

Compared with the liquid phase of the original protein peptide, the foaming property and foam stability of the protein peptide were significantly decreased under the three drying methods. However, the frothiness and foam stability of freeze-dried protein peptide samples were significantly higher than those of vacuum-dried samples and spray-dried samples. When the protein peptide solution of salted duck egg white is stirred by the machine, a lot of gas will be sucked into it, thus creating a water vapor interface. The protein peptide of salted duck egg white is a kind of amphiphilic substance, which can bond between water and atmosphere, thereby reducing surface tension and accelerating the formation of bubbles. The intermolecular binding of egg white protein peptides can enhance the stability of foam (27). The foamability and foam stability of spray-dried samples decreased to 121.79 and 34.83%, respectively. The protein peptide should be soluble for foam formation. The decrease in foaming ability indicated that insoluble molecules were formed by the aggregation of protein peptides during the drying process, which reduced foaming ability and foam stability. Ovalbumin can be modified with glucose molecules to improve its heat-induced aggregation resistance. Aoki et al. (28) used oligo-galacturonic acid to modify ovalbumin through non-enzymatic Browning reaction, and the results showed that the thermal stability of ovalbumin was significantly improved.

### Emulsification analysis under three drying methods

**Figure 4:**
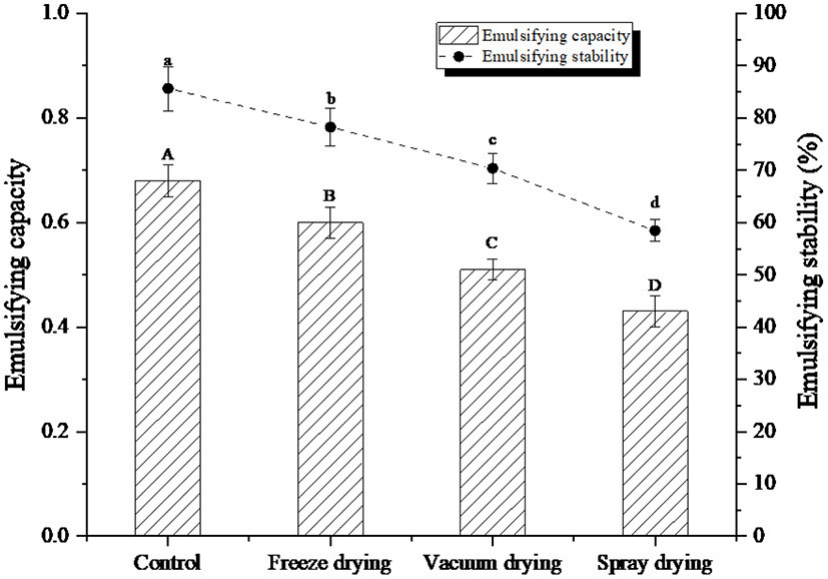
Emulsifying capacity and emulsifying stability of protein peptide powder by three dry methods. Different letters express significant differences (*p* < 0.05).

Emulsification of proteins refers to the ability of proteins to participate in the formation of emulsions, while emulsification stability refers to the ability of emulsion droplets to remain dispersed without fat floating, flocculation, and aggregation within a certain period (29). The emulsification and emulsification stability of the protein peptide of salted duck egg white under three drying methods were shown in Figure 4. After drying, the emulsification and emulsification stability of protein peptides decreased to different degrees. Among them, the emulsification and emulsification stability of freeze-dried samples were the closest to the control samples. However, the emulsification and emulsification stability of spray-dried samples decreased the most, which may be due to the destruction of hydrophilic and hydrophobic groups of protein peptides caused by heating, leading to the degradation of emulsification and emulsification stability of protein peptides.

Salted duck egg white protein is a kind of parental structure, and its molecular structure contains both hydrophilic and hydrophobic structures. In a mixture of oil and water, the egg white protein can form a protective film. The protective film can effectively prevent the accumulation and destruction of oil beads (30). Dickinson (31) found in their study that less molecular aggregation improved the viscosity of the solution system and can maintain the emulsion stability of the solution. In addition, under spray drying and heating, protein molecules re-form aggregated through hydrogen and disulfide bonds and other forces. Aggregation can reduce the flexibility of protein peptide molecules, which was also an important reason for its emulsification and emulsification stability.

### Structural characterization of three drying methods of protein peptide powder

**Figure 5:**
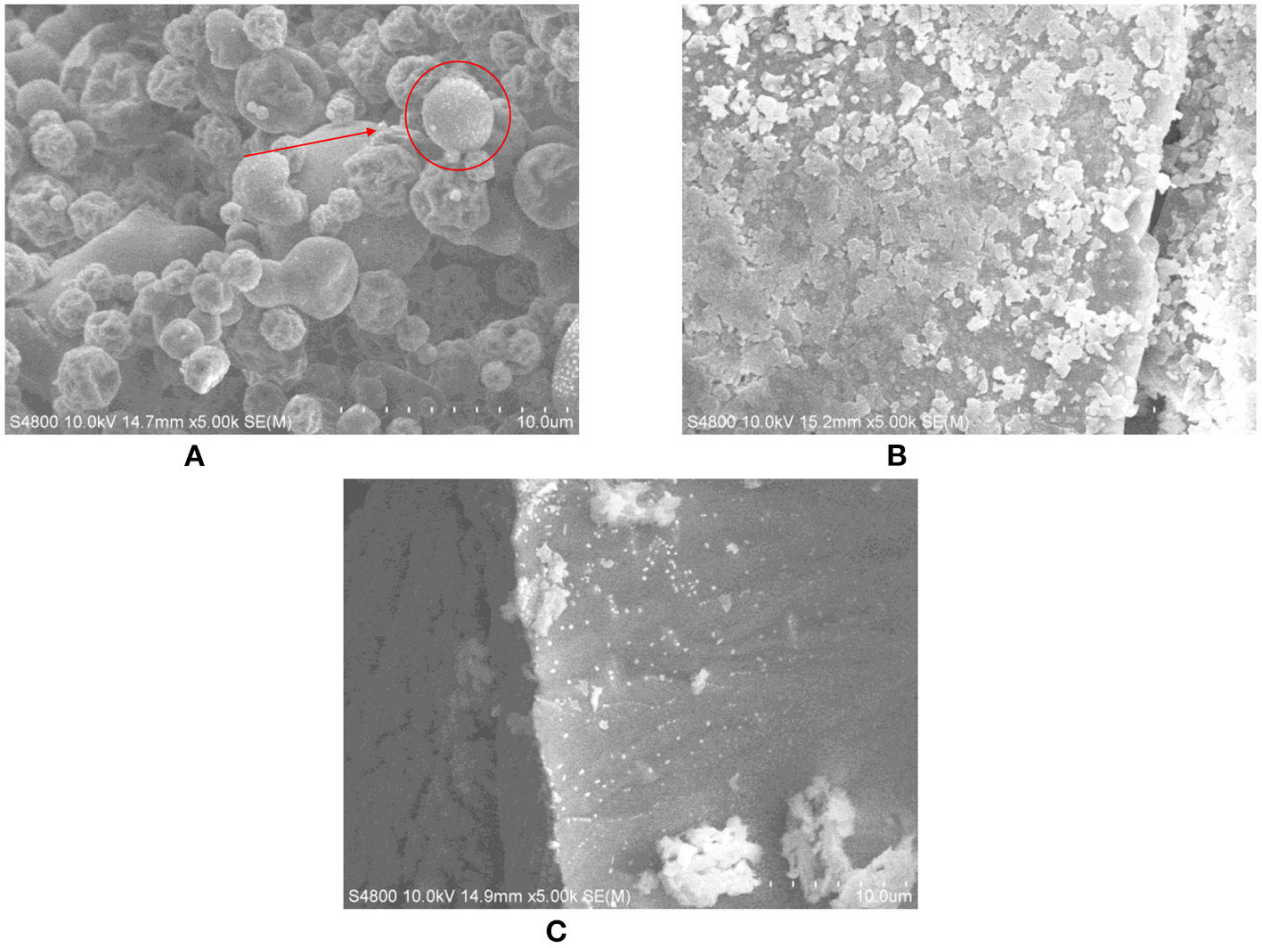
Microstructure of protein peptide powder by three drying methods. **(A)** Freeze drying. **(B)** Spray drying. **(C)** Vacuum drying.

The powder structure of protein peptide powder of salted duck egg white prepared by different drying methods was analyzed by scanning electron microscope (SEM). As can be seen from Figure 5, at 5,000 times magnification, the protein powder of salted duck egg white under freeze-drying treatment was relatively regular. The protein powder structure was close, with local subtle holes, and the texture was full. The size of protein powder particles was round and the surface was glossy. The surface of salted duck egg white protein powder under spray drying treatment was unfolded. The size of the protein powder was uniform. The texture was loose and no longer spherical.

Heat can cause protein molecules to denature and change the secondary structure of the protein. In scanning electron microscopy, the spray-dried protein structure was the most heterogeneous. This may be because the side chain groups buried deep inside the protein molecules were exposed by spray drying. The molecular flexibility was enhanced, and the unfolding and morphological change of the protein molecules at the interface was easier (32). The surface of salted duck egg white protein powder under vacuum drying treatment was poor, and the protein structure become rough and more irregular. In general, freeze-drying had the least effect on the microstructure of protein and peptide powder from salted duck egg white.

### Analysis of superoxide anion (O_2_-·) scavenging ability of protein peptides under three drying methods

Superoxide anion (O_2_-·) belongs to reactive oxygen species and is the precursor of oxygen radical. Superoxide anion has strong oxidation property. Under certain conditions, the superoxide anion can be converted into other free radicals (33). In this experiment, the pyrogallol autoxidation method was used to determine the superoxide anion. Under alkaline conditions, pyrogallol underwent rapid autoxidation to form a semi-quinone radical and superoxide anion. Subsequently, the semi-quinone radical is further oxidized by superoxide anion to form quinone with strong absorption, so the absorbance increases accordingly. The superoxide anion will be quenched in the presence of antioxidant substances in the solution. Semi-quinone radicals cannot form strongly absorbed quinones. Therefore, the antioxidant activity of the sample can be determined by measuring its absorbance.

**Figure 6:**
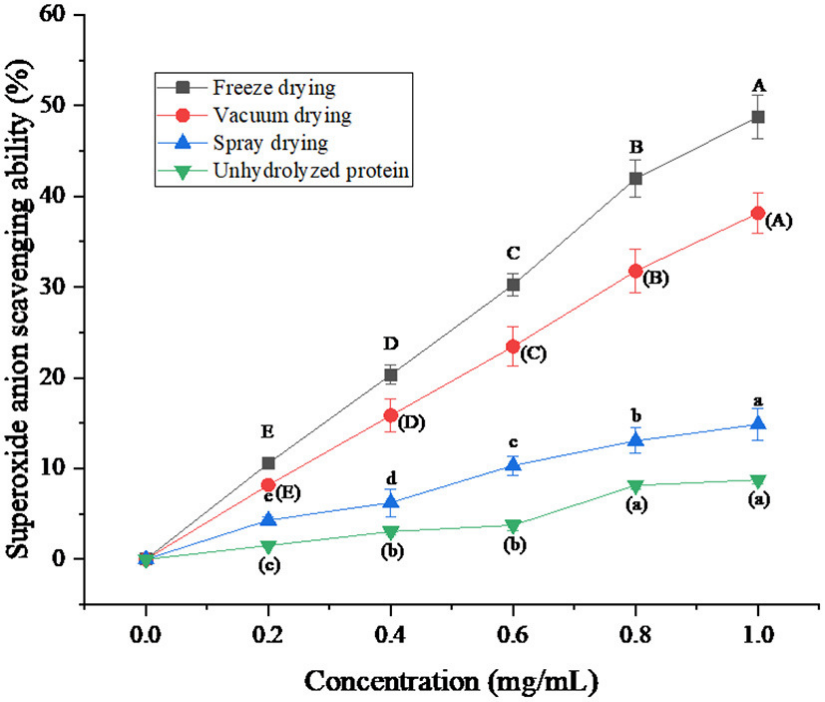
Superoxide anion (O2-·) scavenging ability of protein peptide powder by three dry methods. Different letters express significant differences (*p* < 0.05).

The superoxide anion scavenging ability of protein peptides and unhydrolyzed proteins was compared under three drying methods. As can be seen from Figure 6, the ability of the protein peptide of salted duck egg white to remove superoxide anion increased with the increase of the protein peptide concentration. The scavenging capacity of unhydrolyzed proteins was much lower than that of enzymatic peptides. Niranjan et al. (34) found that the ability of protein peptides to clear superoxide anion was positively correlated with its concentration. The scavenging ability of freeze-dried protein peptides was better than that of vacuum-dried and spray-dried samples. When the concentration of protein peptide was 1 mg/mL, the clearance rate of superoxide anion by freeze-dried protein peptide could reach 48.76%. This indicates that the freeze-drying method to dry the protein peptide of salted duck egg white can effectively ensure the ability of its active peptide to remove superoxide anion.

### Analysis of hydroxyl radical (·OH) scavenging ability of protein peptides under three drying methods

Hydroxyl radical is a highly reactive free radical with rapid reaction and strong attack power, which is more active in cell reaction (35). Hydroxyl radicals react with various molecules in the body mainly through electron transfer, addition reaction, and dehydrogenation, thereby causing oxidative damage to carbohydrates, proteins, nucleic acids, and other substances, and even leading to aging or carcinogenesis (36).

**Figure 7:**
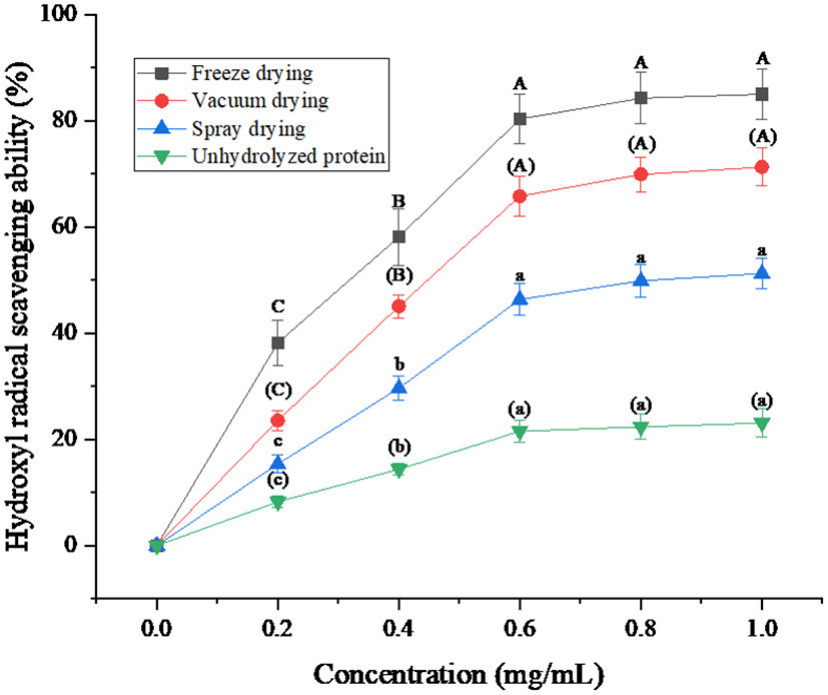
Hydroxyl radical (·OH) scavenging ability of protein peptide powder by three dry methods. Different letters express significant differences (*p* < 0.05).

Hydroxyl radical scavenging rate is an important index to reflect the antioxidant capacity of protein peptides from salted duck egg white. As can be seen from Figure 7, the hydroxyl radical scavenging rate of protein peptides increased with the increase of protein peptide concentration under the three drying methods and was greater than that of unhydrolyzed protein. The hydroxyl radical scavenging ability of freeze-dried protein peptides was better than that of vacuum-dried and spray-dried samples. Sarabandi and Jafari (37) also found that the shear tension and dehydration during spray-drying resulted in instability of the peptides, and reduced the hydroxyl radical scavenging ability of peptides. When the concentration of protein peptide was 1 mg/mL, the hydroxyl radical scavenging rate of freeze-dried protein peptide was up to 85.03%. The experimental results showed that the freeze-drying method to dry the protein peptide of salted duck egg white could effectively guarantee the hydroxyl radical scavenging ability of its active peptide.

### DPPH· scavenging ability analysis of protein peptides under three drying methods

DPPH· free radical is a very stable free radical with a nitrogen center, which is one of the important indicators of the antioxidant capacity of samples (38). The DPPH· radical has one electron and its alcohol solution is purple with strong absorption at 515 nm. In the presence of antioxidants, DPPH· radicals were scavenged, the solution became lighter in color, and the absorbance at 515 nm decreased. The change of absorbance is proportional to the degree of free radical scavenging in a certain range (39).

**Figure 8:**
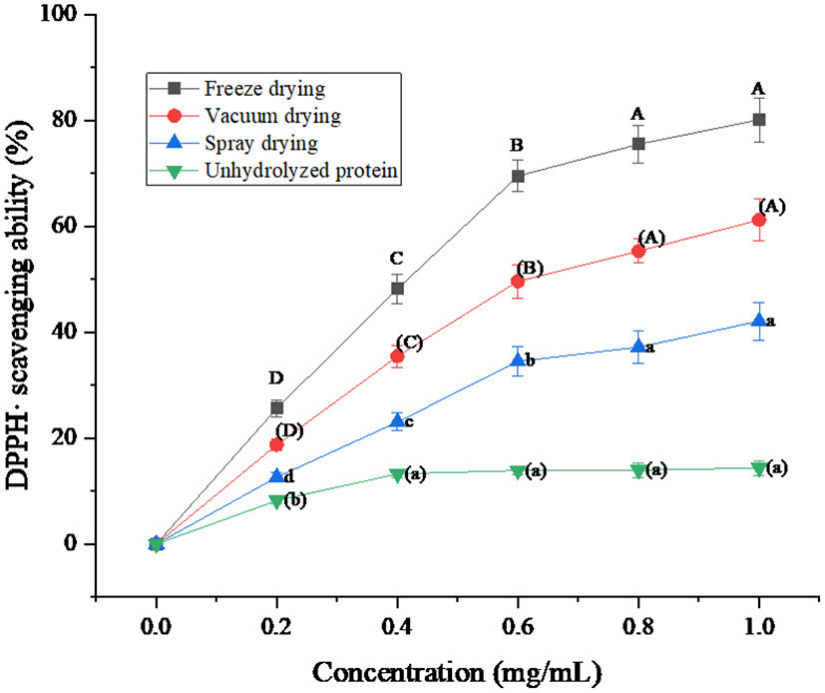
DPPH· scavenging ability of protein peptide powder by three dry methods. Different letters express significant differences (*p* < 0.05).

It can be seen from Figure 8 that the protein peptide of salted duck egg white has a relatively obvious scavenging effect on DPPH·. When the protein concentration was below 0.6 mg/mL, the clearance rate of DPPH· by the protein peptide increased linearly. When the protein concentration was higher than 0.6 mg/mL, the clearance rate of DPPH· by protein peptides began to increase slowly. In addition, it was obvious that the DPPH· scavenging rate of protein peptides under the three drying methods was higher than that of unhydrolyzed protein. The DPPH· scavenging ability of freeze-dried protein peptides was better than that of vacuum-dried and spray-dried samples. When the protein peptide concentration was 1 mg/mL, the clearance rate of DPPH· by freeze-dried protein peptide could reach 80.17%. Foh et al. (40) hydrolyzed tilapia freeze-dried protein powder to obtain peptides. Then the DPPH· scavenging ability of the peptides was determined. The results showed that the scavenging ability of DPPH· by tilapia peptides was as high as 86.67%. It can be concluded that the protein peptide of salted duck egg white dried by the freeze-drying method has a higher antioxidant capacity than that of unhydrolyzed salted egg white.

## Conclusions

The physicochemical properties and antioxidant capacity of the protein peptide powder of salted duck egg white prepared by different drying methods were studied in this paper. The results showed that under the same conditions, the physicochemical properties and antioxidant activity of protein peptide powder of salted duck egg white could be maintained better by freeze-drying, while the effect of spray drying was the worst. After drying in different ways, the solubility, foaming and foaming stability, emulsification and emulsification stability of the protein peptide of salted duck egg white decreased to different degrees. Among them, the samples dried by freeze-drying had the lowest decrease. While the air inlet temperature of spray drying (180°C) was higher, the solubility, foaming and foaming stability, emulsification and emulsification stability of samples by spray drying decreased the most. This may be due to the low temperature of freeze drying, which is not easy to cause protein denaturation. The spatial structure of protein peptide was changed by drying, and the physicochemical properties and antioxidant activity of protein peptide powder were changed.

The scavenging rates of superoxide anion, hydroxyl radical, and DPPH· in the protein peptide of salted duck egg white were decreased by different drying methods. Freeze-dried samples showed the smallest decrease. When the concentration of protein peptide was 1 mg/mL, the scavenging rates of superoxide anion, hydroxyl radical, and DPPH· of freeze-dried samples were 48.76, 85.03, and 80.17%, respectively. The microstructure analysis of protein peptide of salted duck egg white showed that the protein powder of salted duck egg white under freeze-drying treatment was regular and the protein powder particles were round. The protein particles of salted duck egg white were no longer spherical under spray drying. The results showed that freeze-drying was an ideal drying method for the protease hydrolysate of salted duck egg white.

## Data availability statement

The raw data supporting the conclusions of this article will be made available by the authors, without undue reservation.

## Author contributions

TD, SZ, and XY: writing—original draft. JX, JG, and WL: writing—review and editing. All authors contributed to the article and approved the submitted version.

## Funding

The authors acknowledged the support of the Anhui Provincial Science and Technology Major Special Project (202003b06020004), the Anhui Provincial Natural Science Foundation (No. 1908085MC79), and the Overseas Visiting and Study Program for Outstanding Young Backbone Talents of Anhui Universities (No. gxgwfx2020056).

## Conflict of interest

The authors declare that the research was conducted in the absence of any commercial or financial relationships that could be construed as a potential conflict of interest.

## Publisher's note

All claims expressed in this article are solely those of the authors and do not necessarily represent those of their affiliated organizations, or those of the publisher, the editors and the reviewers. Any product that may be evaluated in this article, or claim that may be made by its manufacturer, is not guaranteed or endorsed by the publisher.
